# Evaluating preferences for colorectal cancer screening in individuals under age 50 using the Analytic Hierarchy Process

**DOI:** 10.1186/s12913-021-06705-9

**Published:** 2021-07-29

**Authors:** Travis Hyams, Bruce Golden, John Sammarco, Shahnaz Sultan, Evelyn King-Marshall, Min Qi Wang, Barbara Curbow

**Affiliations:** 1 Department of Behavioral and Community Health, School of Public Health, University of Maryland, College Park, USA; 2 Division of Cancer Control and Population Sciences, Office of the Director, National Cancer Institute, Bethesda, USA; 3 Department of Decision, Operations, and Information Technologies, Robert H. Smith School of Business, University of Maryland, College Park, USA; 4 Definitive Business Solutions, Inc., 11921 Freedom Drive, Suite 550, Reston, VA 20190 USA; 5 Department of Medicine, Division of Gastroenterology, Hepatology, and Nutrition, University of Minnesota, Minneapolis, USA

**Keywords:** Colorectal cancer, Screening, Multicriteria decision analysis, Preferences, Medical decision making

## Abstract

**Background:**

In 2021, the United States Preventive Services Task Force updated their recommendation, stating that individuals ages 45-49 should initiate screening for colorectal cancer. Since several screening strategies are recommended, making a shared decision involves including an individual’s preferences. Few studies have included individuals under age 50. In this study, we use a multicriteria decision analysis technique called the Analytic Hierarchy Process to explore preferences for screening strategies and evaluate whether preferences vary by age.

**Methods:**

Participants evaluated a hierarchy with 3 decision alternatives (colonoscopy, fecal immunochemical test, and computed tomography colonography), 3 criteria (test effectiveness, the screening plan, and features of the test) and 7 sub-criteria. We used the linear fit method to calculate consistency ratios and the eigenvector method for group preferences. We conducted sensitivity analysis to assess whether results are robust to change and tested differences in preferences by participant variables using chi-square and analysis of variance.

**Results:**

Of the 579 individuals surveyed, 556 (96%) provided complete responses to the AHP portion of the survey. Of these, 247 participants gave responses consistent enough (CR < 0.18) to be included in the final analysis. Participants that were either white or have lower health literacy were more likely to be excluded due to inconsistency. Colonoscopy was the preferred strategy in those < 50 and fecal immunochemical test was preferred by those over age 50 (*p* = 0.002). These results were consistent when we restricted analysis to individuals ages 45-55 (*p* = 0.011). Participants rated test effectiveness as the most important criteria for making their decision (weight = 0.555). Sensitivity analysis showed our results were robust to shifts in criteria and sub-criteria weights.

**Conclusions:**

We reveal potential differences in preferences for screening strategies by age that could influence the adoption of screening programs to include individuals under age 50. Researchers and practitioners should consider at-home interventions using the Analytic Hierarchy Process to assist with the formulation of preferences that are key to shared decision-making. The costs associated with different preferences for screening strategies should be explored further if limited resources must be allocated to screen individuals ages 45-49.

**Supplementary Information:**

The online version contains supplementary material available at 10.1186/s12913-021-06705-9.

## Background

### Colorectal Cancer and screening guidelines

Colorectal-cancer (CRC) related incidence and mortality is declining in the United States and across the globe, primarily due to public health efforts to improve age appropriate, guideline-based screening. However, it still remains a leading cancer control priority [1–4]. Recent analyses indicate an alarming trend of increasing risk in individuals under age 50 [4, 5]. In 2018, the American Cancer Society updated it’s guidelines for colorectal cancer screening to address this trend in younger individuals and made a ‘qualified’ recommendation that average-risk adults aged 45 or older should begin regular screening [1]. In May of 2021, the United States Preventive Services Task Force (USPSTF), an independent guideline-producing organization whose recommendations influence clinical coverage and policy decisions, released their updated recommendation statement to initiate screening at age 45 [6]. This shift will add approximately 19 million individuals to the eligible CRC screening pool [7].

### Preferences for screening strategies

There are several effective but different CRC screening strategies that fall broadly into either stool-based strategies or structural, visual exams and are conducted at varying screening intervals. The effectiveness and characteristics of individual screening strategies are discussed in Wolf, et al. (2018) [1]; and Lin, et al. (2016) [8]. These strategies are all recommended but differ with respect to characteristics that could influence an individual’s preference and adherence to that screening strategy. To ensure uptake of recommendations, the choice of screening strategy should depend on the individual’s preferences, test availability, and the strategy that the patient is most likely to complete and adhere to [1, 6, 9]. The existing literature on preferences for screening strategies shows high variability in preferences across studies [10–13] and, since guidelines have previously recommended screening at age 50, few studies have included younger people or explicitly tested differences in preferences by age.

### The Analytic Hierarchy Process and colorectal cancer screening

Multicriteria Decision Analysis (MCDA) techniques can be used to help individuals make complex decisions. As a research tool, MCDA is a valuable means to explore underlying preferences on which a decision is based. In a clinical setting, MCDA can facilitate decision-making processes and encourage informed and shared decision making by helping patients think critically about all available options and their unique characteristics. The Analytic Hierarchy Process (AHP) is a MCDA technique that breaks a decision problem into a hierarchical structure and allows a decision maker to focus on one aspect of a complex decision at a time [14]. The AHP also enables one to quantify preferences for decision alternatives and the criteria on which those preferences are based. The AHP technique can be used in healthcare decision making research and practice [15] in areas such as shared decision making, healthcare policy and evaluation, and human resource planning [16]. Individuals are generally willing and able to use AHP for decision making [17, 18]. AHP can be used for individual level decisions and results across individuals can be aggregated into a group decision. Aggregating to the group level allows one to compare hierarchy weights across different subgroups to determine if they are evaluating the hierarchy differently.

Several studies have used AHP to assess preferences for colorectal cancer screening strategies [18–20]. These studies were successful in the implementation of AHP and found that the screening strategy’s effectiveness or ability to detect and prevent cancer was paramount in the decision-making process. Only Xu (2015) and colleagues included individuals under age 50 (mean age of the participants in their analytic sample was 56.7 years old) but they did not conduct age subgroup analysis to assess differences in preferences by age [19].

### Current study

Current guidelines suggest that any evidence-based screening strategy is better than an individual not getting screening and that individuals generally have preferences for CRC screening strategies when given a choice [10]. Therefore, preferences should be assessed and incorporated into the decision-making process to ensure that individuals are receiving preference-aligned care. Research that unravels these preferences will give providers information to target clinical interactions to the most important issues for younger people. If differences are found, we may consider adapting screening programs for younger populations to ensure that they are satisfied with their decision.

In the current study, we used the AHP to quantify preferences for colorectal cancer screening strategies and the criteria on which these preferences are based. We also assess whether preferences are related to any key characteristics of participants including health literacy and previous experience with cancer.

## Methods

### Study sample

This study was part of a larger study on perceptions about colorectal cancer in individuals under age 50. We collected a convenience sample whereby participants self-selected to participate from Amazon Mechanical Turk (MTurk). MTurk describes itself as a crowdsourcing marketplace that leverages a global workforce to complete a variety of tasks, including research tasks [21]. MTurk workers (Turkers) represent diverse individuals from across the globe and previous work with MTurkers has shown that they produce high quality research data, even when presented with complex behavioral tasks that are traditionally conducted in-person [22]. We took best-practice measures to ensure high quality data including requiring workers to have completed tasks with a high approval rating and including qualitative responses to screen for automated responses.

We conducted a pilot test with 30 individuals ages 45-55 prior to the launch of the full survey to ensure adequate understanding of the questions and procedures. Due to minor changes in survey procedures from the pilot test, these individuals were not included in the final analytic sample. Initial inclusion criteria for the final launch included being ages 45-55, living in the United States, and never having been screened for colorectal cancer. After an initial collection of 482 participants, we then released the survey to all individuals over 18 who met these criteria. Survey data were collected using Qualtrics [23].

### Hierarchy development

**Fig. 1:**
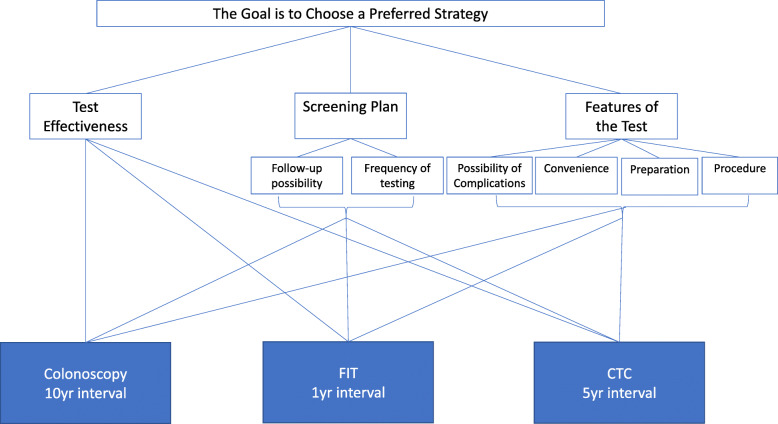
Hierarchy structure

In this paper, screening strategy is defined as the test, the subsequent follow-up to an abnormal result, and the regular screening interval for normal results. In subsequent references, criteria will be in **bold,** sub-criteria will be *italicized*. The AHP hierarchy (Fig. 1) was built to include 3 criteria, 6 sub-criteria, and 3 decision alternatives (screening strategies). Criteria and sub-criteria were selected based on a review of prior AHP studies in the colorectal cancer screening literature [18–20]. An initial model was developed by the lead author (TH) and study investigators including a gastroenterologist (SS), AHP methodologists (BG, JS), and subject matter experts (E K-M, & BC) discussed the hierarchy and came to a group consensus on the final model.

Criteria included **test effectiveness,** the **screening plan,** and **features of the test**. The **screening plan** includes 2 sub-criteria: the *follow-up possibility* and *frequency of testing*. **Features of the test** included 4 sub-criteria: the *possibility of complications, convenience,* the *preparation,* and the *procedure*. Similar to other AHP studies in the literature [18–20], we did not include out-of-pocket cost in our hierarchy because it would be impossible to present individualized cost estimates for every participant. We presented 3 distinct screening strategies as decision alternatives: colonoscopy every 10 years (an invasive test that allows for direct visualization of the colon), fecal immunochemical test (FIT) every year (a stool-based test that detects blood in the stool) and computed tomography colonography (CTC) every 5 years (a radiographic test). All screening strategies and intervals that we have selected for this study are recommended by the USPSTF [6].

### AHP assessment

Survey procedures were approved by the University of Maryland IRB. All survey procedures were self-guided, and participants completed questions at their own pace. After completing demographic and knowledge survey items, participants read a brief overview of colorectal cancer and the importance of screening (Supplemental materials 1). They were also informed that the goal of the activity was to choose a preferred screening strategy. Participants then read statements (Supplemental materials 1) about each strategy related to each criteria/sub-criteria at the lowest level of the hierarchy.

After each description, we asked participants to rank the decision alternative with respect to that criteria from (1) Does not fit to (9) Fits extremely well: *“*When considering the *(***Criteria***/Sub-criteria): On a scale from 1 - 9,* how well does (Decision Alternative) fit your preferences?” Overall, participants made 3 × 7 = 21 direct comparisons for the 3 decision alternatives and 7 criteria/sub-criteria (**Test effectiveness**, *follow-up possibility, frequency of testing, possibility of complications, convenience, preparation,* and *procedure*). We used direct comparisons on decision alternatives to reduce respondent burden and eliminate the risk of rank reversal [14]. We presented direct comparisons in randomized order using the Qualtrics question randomization feature.

Participants then made pairwise comparisons at each level of the hierarchy to assess which criteria/sub-criteria was more important for their decision: *“*When considering the *(***Criteria/***Sub-criteria):* Which is more important for your decision?*”* Participants were given the option to select “**Criteria**/*Sub-criteria 1*, **Criteria**/*Sub-criteria 2* or ‘They are both equal’”. Participants then ranked their selection on a scale from 1 (very slightly more important) to 9 (extremely more important). To assess stated preferences, after the AHP procedure, participants were asked: “After completing this exercise, which test for colorectal cancer would you choose?”. This study evaluated all participants that provided complete data for the AHP portion of our survey. We did not conduct a power analysis for this study because the AHP is appropriate for individuals or groups of any size.

### Independent variables

Demographic variables included age (1 = < 50, 2 = ≥50); education (1 = high school or less, 2 = some college, 3 = college completion, 4 = higher than college); household income (1 = < 30 k, 2 = 30 k to 59,999, 3 = 60 k to 89,999, 4= > 90 k); gender identity (1 = man, 2 = woman), self-identified primary race (recoded as 1 = white, 2 = other); type of insurance coverage (recoded as 0 = no, 1 = public, 2 = private), relationship status (recoded as 1 = single, 2 = dating or cohabitating, but not married, 3 = married). We also assessed health literacy [24] (continuous 4-20); subjective numeracy [25] (continuous 1-6); decisional self-efficacy (DSES, continuous 0-100), and having a regular medical provider (0 = no, 1 = yes). Finally, we explored variables related to cancer experience: having somebody close who has died of cancer (0 = no, 1 = yes); and family, spouse, or anybody else close having been tested for CRC (recoded 0 = no/don’t know, 1 = yes).

#### Statistical analysis

We used the pairwise comparisons at each level of the hierarchy to compute reciprocal matrices for each set of comparisons. We then used the eigenvector method, which relies on the matrices principal eigenvector [26] to calculate a ratio scale of priorities for criteria/sub-criteria. Weight estimates are calculated by solving the equation: $A\bullet \hat{w}=\lambda max\bullet \hat{w}$ where A is the matrix of pairwise comparisons elicited from the participant, $\hat{w}$ is its right eigenvector, and *λmax* is the largest eigenvalue of A. We calculated consistency ratios at the criteria level using the Alonso and Lamata linear fit method to evaluate how consistently participants were making judgments [27]. Lower consistency ratios represent more consistent judgments. Participants with consistency ratios (CR) higher than 0.18 were excluded from our AHP analysis. To calculate aggregated group decisions for criteria and sub-criteria, we used the row geometric mean method (RGMM). We assigned participants their individual preferences for decision alternatives based on the greatest of the 3 normalized priority weights for decision alternatives. We performed Chi-squared for categorical variables and ANOVA for continuous variables to determine associations. We conducted sensitivity analysis by varying the weights of the criteria and sub-criteria to assess whether observed alternative weightings are sensitive to small changes in the group weighting factors. We conducted statistical analysis using SPSS [28]. For AHP analysis, data were imported into Definitive Pro® [29], a software product designed to help decision-makers comprehensively and consistently assess and prioritize alternatives.

## Results

Of the 579 individuals surveyed, 556 (96%) provided complete responses to the AHP portion of the survey. Of these, 247 (44.4%) participants gave responses consistent enough (CR < 0.18) to be included in the final analysis. The demographic features for included and excluded participants can be found in Table 1.

**Table 1:** Participant demographic characteristics

	**Included Subjects** **(CR < .18)** ***N*** **= 247** **N (%)**	**Excluded Subjects** **(CR > .18)** ***N*** **= 309**	***p*-values χ2**
**Categorical Variables**			
**Age**			
< 50	160 (65)	190 (61.5)	0.389
50+	86 (35)	119 (38.5)	
**Gender**			
Woman	157 (63.6)	182 (58.9)	0.256
Man	90 (36.4)	127 (41.1)	
**Race**			
White / Caucasian	198 (80.2)	268 (86.7)	0.037
Not white/Caucasian	49 (19.8)	41 (13.3)	
**Education categories**			
HS or less	45 (18.2)	51 (16.6)	0.474
Some college	65 (26.3)	88 (28.6)	
College graduate	88 (35.6)	95 (30.8)	
Some graduate school / Graduate degree	49 (19.8)	74 (24.0)	
**Relationship**			
Single	93 (37.7)	110 (35.8)	0.769
Relationship / cohabitating	35 (14.2)	50 (16.3)	
Married	119 (48.2)	147 (47.9)	
**Income**			
< 30 k	52 (21.1)	54 (17.5)	0.472
30 k to 59,999	72 (29.3)	102 (33)	
60 k to 89,999	63 (25.6)	70 (22.7)	
> 90 k	59 (24)	83 (26.9)	
**Insurance**			
No	38 (15.4)	48 (15.7)	0.751
Private	166 (67.2)	212 (69.3)	
Public	43 (17.4)	46 (15.0)	
	**Mean (SD)**	**Mean (SD)**	**ANOVA**
**Continuous Variables**			
**Health literacy**	18.15 (2.2)	17.63 (2.7)	0.014
**Subjective numeracy**	4.59 (.97)	4.67 (.90)	0.302
**Decisional self-efficacy**	82.2 (16)	80.0 (15.9)	0.107

**Fig. 2:**
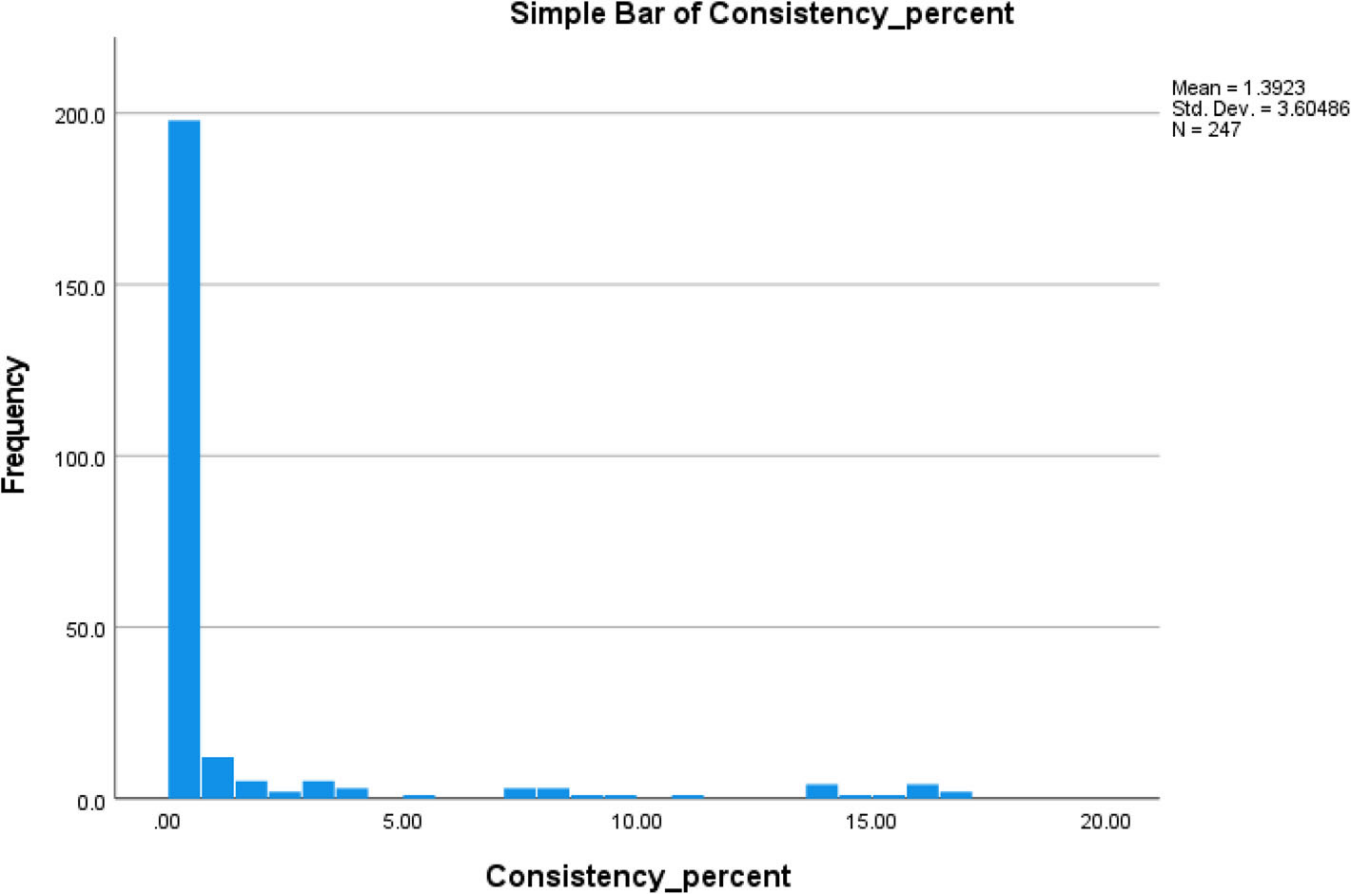
Consistency ratios of included participants

Participants who are either white or have lower health literacy were more likely to be excluded from the final analysis due to inconsistency. The spread of consistency ratios < 0.18 can be found in Fig. 2. The mean age of included participants in the < 50 group was 45 years old and in the 50+ age group the mean age was 52.5. Included participants in the two age subgroups were similar on demographic variables including gender, self-identified race, education, relationship status, income, insurance, health literacy, subjective numeracy, and decisional self-efficacy.

### Preferences for screening strategy (decision alternatives)

#### Group decision

**Fig. 3:**
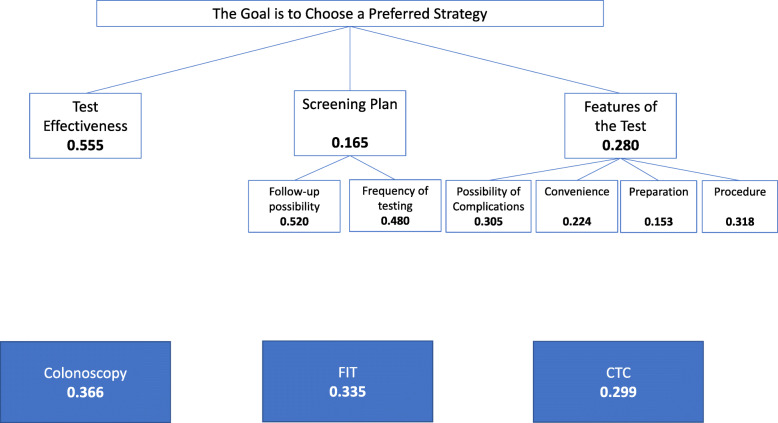
Group decision weighting factor results *N* = 247

We used the group decision to compare hierarchies in the overall sample and in each age subgroup using the row geometric mean. The aggregated preference for the whole sample (Fig. 3, *n* = 247) was for colonoscopy, with a normalized preference of 0.366, followed by FIT (0.335) and CTC (0.299). In the < 50 group (*n* = 161), colonoscopy was the preferred test (0.375) followed by FIT (0.321) and CTC (0.304). In the ≥50 (*n* = 86), the group preference was for FIT (0.366) followed by colonoscopy (0.345) and CTC (0.289).

#### Individual preferences

To assess whether the differences we found at the group level were driven by participant variables, we assessed individual level preferences for screening tests (Table 2). The AHP procedure revealed preferences for 239 participants. Eight participants (3.2%) scored equally for multiple screening tests or did not prefer any of the tests. Of the participants who had preferences, colonoscopy was preferred by 50.6%, 40.6% preferred FIT, and 8.8% preferred CTC. In the < 50 age group, colonoscopy was the preferred screening test by 57.7% of participants while, in the ≥50 age group, most participants (56.1%) preferred FIT. Chi-square tests revealed significant differences between the proportion of individuals who preferred colonoscopy and FIT in the two age groups (*p* = 0.002). These results were consistent when we restricted the analysis to those ages 45-55 (*n* = 208). In the 45-49 age group, 58.3% preferred colonoscopy, while in the 50-55 age group, 55.6% preferred FIT (*p* = 0.011). Those with lower health literacy (*p* = 0.002) and lower decisional efficacy (*p* = 0.041) were more likely to select the CTC option compared to colonoscopy or FIT. We did not find associations between test selection and any other demographic variables in our sample. The majority (59.8%) of participants stated preferences that were concordant with their AHP-derived preferences (Supplemental Materials 2).

**Table 2:** Preferences for screening strategies from AHP

	**Colonoscopy** **N (%)**	**CTC** **N (%)**	**FIT** **N (%)**	***p*-value χ2**
Full Sample with preference (*N* = 239)	121 (50.6)	21 (8.8)	97 (40.6)	
	N(%)	N(%)	N(%)	
**Categorical Variables**				
**Age**				
< 50	90 (57.7)	15 (9.6)	51 (32.7)	0.002
50+	31 (37.8)	5 (6.1)	46 (56.1)	
**Gender**				
Woman	73 (48.3)	10 (6.6)	68 (45)	0.100
Man	48 (54.5)	11 (12.5)	29 (33)	
**Race**				
White/Caucasian	100 (51.5)	14 (7.2)	80 (41.2)	0.205
Not white/Caucasian	21 (46.7)	7 (15.6)	17 (37.8)	
**Education**				
HS or less	19 (43.2)	7 (15.9)	18 (40.9)	0.581
Some college	33 (52.4)	3 (4.8)	27 (42.9)	
College graduate	42 (50.6)	7 (8.4)	34 (41)	
Some graduate school / Graduate degree	27 (55.1)	4 (8.2)	18 (36.7)	
**Relationship**				
Single	41 (45.6)	10 (11.1)	39 (43.3)	0.396
Relationship/cohabitating	15 (42.9)	3 (8.6)	17 (48.6)	
Married	65 (57)	8 (7)	41 (36)	
**Income**				
< 30 k	25 (50)	3 (6)	22 (44)	0.548
30 k to 59,999	30 (44.1)	5 (7.4)	33 (48.5)	
60 k to 89,999	32 (52.5)	8 (13.1)	21 (34.4)	
> 90 k	33 (55.9)	5 (8.5)	21 (35.6)	
**Insurance**				
No	16 (45.7)	1 (2.9)	18 (51.4)	0.277
Private	88 (54)	15 (9.2)	60 (36.8)	
Public	17 (41.5)	5 (12.2)	19 (46.3)	
**Regular provider**				
Yes	79 (54.5)	13 (9.0)	53 (36.6)	0.276
No	42 (44.7)	8 (8.5)	44 (46.8)	
**Family, spouse, other close tested for CRC**				
Yes	86 (51.8)	12 (7.2)	68 (41)	0.434
No	35 (47.9)	9 (12.3)	29 (39.7)	
**Anybody close ever died of cancer**				
Yes	76 (56.7)	11 (8.2)	47 (35.1)	0.099
No	45 (42.9)	10 (9.5)	50 (47.6)	
	**Mean (SD)**	**Mean (SD)**	**Mean (SD)**	**ANOVA**
**Continuous Variables**				
**Health literacy**	18.2 (2.0)	16.6 (3.1)	18.4 (2.0)	0.002
**Numeracy**	4.6 (.96)	4.2 (1.0)	4.6 (.98)	0.205
**Decisional self-efficacy**	82.9 (15.5)	74 (17.4)	83.6 (15.4)	0.041

Individuals that expressed no clear preference (*n* = 8) were excluded from this analysis

### Criteria and sub-criteria

As a group (Fig. 3, *n* = 247), the sample weighed **test effectiveness** as the most important criteria for making their decision (0.555) followed by the **features of the test** (0.280) and the **screening plan** (0.165). For the sub-criteria of the **screening plan**, they rated *frequency of testing* and the *possibility of follow-up as* similarly important, at 0.480 and 0.520 respectively. For **features of the test***,* the *procedure* was weighted as most important (0.318), followed by the *possibility of complications* (0.305), the *convenience* (0.224) and the *preparation* (0.153).

In the under 50 and ≥ 50 age groups, the relative priority order was similar to the larger group for the criteria (**effectiveness, screening plan, features**) and sub-criteria of **features of the test** *(preparation, convenience, complications, procedure).* For the sub-criteria of **screening plan***,* the under 50 group weighed *possibility of follow-up* (0.499) and *frequency* (0.501) almost equally, while the ≥50 age group weighed the *possibility of follow-up* (0.548) as slightly more important than *frequency of testing* (0.452). Even though the relative priority order was similar between groups, and **test effectiveness** was most important for both groups, we found differences in the magnitude of priorities. The under 50 age group assigned a relative priority of 0.585 to **test effectiveness** while the ≥50 age group assigned it 0.495. The ≥50 age group assigned higher importance to the **features of the test** (0.340) than younger people (0.250). Both age groups rated **screening plan** at approximately 0.165. The priority results did not change when we restricted the analysis to the 45-55 age group.

#### Ratings of tests by criteria

The results of direct comparisons of screening strategies by criteria/sub-criteria can be found in Table 3. The group (*n* = 247) rated colonoscopy as highest for the criteria **test effectiveness** and **screening plan**, while they rated FIT as highest for the criteria **features of the test**. For the sub-criteria of **screening plan**, colonoscopy was rated most favorably for both *follow-up* and *frequency*. For all 4 sub-criteria of **features of the test** (*complications, convenience, preparation,* and *procedure*), FIT was rated highest. The highest rated criteria/sub-criteria were stable when the sample was split by age.

**Table 3:** Normalized priorities of screening strategies by criteria/sub-criteria

	Overall *n* = 247	Under 50 *n* = 161	50+ *n* = 86
**Test effectiveness**			
Colonoscopy	**0.417**	**0.415**	**0.421**
FIT	0.284	0.281	0.292
CTC	0.299	0.304	0.287
**Screening Plan**			
Colonoscopy	**0.425**	**0**.**436**	**0.403**
FIT	0.279	0.263	0.310
CTC	0.296	0.301	0.287
*Follow up*			
Colonoscopy	**0.456**	**0.468**	**0.432**
FIT	0.277	0.262	0.308
CTC	0.267	0.270	0.260
*Frequency*			
Colonoscopy	**0.397**	**0.410**	**0.372**
FIT	0.280	0.264	0.312
CTC	0.323	0.326	0.316
**Features of the test**			
Colonoscopy	0.218	0.230	0.197
FIT	**0.481**	**0.464**	**0.513**
CTC	0.301	0.306	0.290
*Complications*			
Colonoscopy	0.200	0.210	0.179
FIT	**0.520**	**0.505**	**0.554**
CTC	0.280	0.285	0.267
*Convenience*			
Colonoscopy	0.176	0.185	0.156
FIT	**0.488**	**0.477**	**0.508**
CTC	0.336	0.338	0.336
*Procedure*			
Colonoscopy	0.266	0.279	0.241
FIT	**0.413**	**0.393**	**0.453**
CTC	0.321	0.328	0.306
*Preparation*			
Colonoscopy	0.217	0.228	0.196
FIT	**0.548**	**0.535**	**0.572**
CTC	0.235	0.237	0.232

#### Sensitivity analysis

From our prior experience and the colorectal cancer literature, we identified bowel preparation as an important barrier to completion of structural colorectal cancer screening exams for some individuals [30]. To address this, we tested whether increasing the weight of preparation would change the weights of decision alternatives. In the overall group, we increased the priority of *preparation* from 0.153 to 0.400 and subsequently, *complications* was reweighted from 0.305 to 0.220, *convenience* from 0.224 to 0.160, and *procedure* from 0.318 to 0.220. These changes had no impact on the order of alternative preferences. In fact, they only changed normalized priority scores of each alternative by < ± .5%. We also tested whether our findings were robust to small changes in weighting factors at the criteria level. When we tested a 5% reduction in **test effectiveness** (from 0.555 to 0.505), with a prorated distribution to the weightings of **features of the test** and **screening plan,** we saw no changes in the rank order of alternatives and minimal (< ±0.5%) changes in the normalized priority scores of alternatives.

## Discussion

Based on our study, colonoscopy was the preferred screening strategy in our overall sample, but we found differences in preferences for screening strategies based on age subgroups. Generally, colonoscopy was the preferred strategy for individuals under 50 and FIT was preferred by those 50 and over, and these results held when analysis was restricted to the 45-49 and 50-55 groups. To our knowledge, the only study in the literature that explicitly tested preferences by age under 50 was conducted by DeBourcy and colleagues in 2008 [31]. In their supermarket sample, they observed a nonsignificant (*p* = 0.12) but illustrative trend in preferences for colonoscopy versus FOBT (another stool based test) by age that are similar to the results of this study. In the under 50 age group, 44.9% of people in their sample preferred colonoscopy, while 49% ages 50-64, and 63.6% ages 65-79 preferred colonoscopy. The differences that we found between those ≥50 and people < 50 in this sample are explained by the relative priority assignment to the **test effectiveness** and **features of the test** criteria**.** Both age groups rated **test effectiveness** as most important for making their decision, however, the ≥50 age group gave **features of the test** higher priority than the < 50 age group. This difference gave FIT a higher priority score than colonoscopy when we applied the criteria weighting factors in the ≥50 age group. Other studies that have used AHP to explore preferences for colorectal cancer screening strategies have similarly found that the test effectiveness is the most important criteria for participant’s decisions. Researchers across studies have conceptualized and operationalized this criteria in slightly different ways (i.e., preventing cancer [18], test accuracy [19], and sensitivity/specificity [20]).

We also found that CTC was preferred more often by participants who had lower levels of literacy and decisional efficacy. This may be explained by these participants defaulting to the ‘middle’ option (between colonoscopy and FIT) when unsure about **effectiveness**, **features,** and the **screening strategy**. We did not find differences in AHP derived preference in our sample by any other demographic or cancer experience variables. These results should be considered in the context of previous literature evaluating preferences for screening strategies, which are variable across studies. Investigators in some studies have found differences in CRC screening strategy preference by race [10], income [32], sex/gender [19, 33], education [19, 32, 33], and family history of CRC [32], among other sociodemographic variables, while other studies have similarly found no significant differences [34, 35]. The variability in the results of these studies is likely due to the population that they recruit including their prior experience with CRC screening, variability in the descriptions of each strategy that are presented to participants, and the screening strategies that the researchers provide as options [10, 34, 35]. Clinical settings should carefully consider if differences in preferences of the patient populations that they serve could influence screening programs and investigators should evaluate these differences when interpreting the results of their research.

Approximately 60% of participants gave a stated preference that was concordant with their AHP derived preference, after completing the AHP exercise. This is similar to what Xu, et al. found (0.57) in their 2015 study on individuals that had completed both colonoscopy and FIT tests [19]. The AHP procedure is not created for or intended to replace traditional decision-making processes but to provide additional information that can be used to facilitate the process of thinking through complex decisions. In a healthcare setting, this process would enable patients to come to a clinic appointment better informed, more ready to ask important questions, and ultimately, make a decision that they are satisfied with [14]. For the remaining 40% of participants who provided discordant preferences, there could be criteria that we did not include in the hierarchy or other beliefs about the screening strategies that influenced participants’ judgments that were otherwise not factored into our AHP model, and these warrant further investigation.

We add to the AHP literature that explores colorectal cancer screening [18–20] in the following unique ways: 1) we recruited people < 50 years and ≥ 50 years old and tested younger age as a predictor of screening strategy preferences; 2) we recruited a United States based national sample that was not based solely on clinic patients; 3) our results offer insight into criteria preferences for individuals under 50 that is not explored elsewhere in the literature; 4) we conducted sensitivity analysis to determine if results were robust to changes at the criteria and sub-criteria level; 5) we tested whether individual preferences were associated with participant variables; and 6) we developed and tested a new hierarchy model that can be considered when incorporating AHP into colorectal cancer screening decision making.

### Strengths and limitations

While there is the unavoidable potential for bias stemming from the descriptions that we developed for each screening strategy, a strength of this study is that we were careful to reduce confounding factors that could act to influence an individual’s decision-making process. We included only participants that have never been screened, we randomized the order of direct comparisons, we ensured consistency in the length and reading level of descriptions, and we used a parallel structure for test characteristics that we described for each strategy (Supplemental materials 1). Using the MTurk platform also allowed us to gather data from a national sample representing diverse perspectives. However, convenience sampling through the MTurk platform is not representative of an average community sample and our sample lacked racial and ethnic diversity. Future studies using targeted or representative sampling methods can be used to assess whether the associations we found remain stable.

Participants also responded to pairwise comparisons with a high level of inconsistency leading to many participants with high consistency ratios warranting exclusion. High consistency ratios are seen in all current AHP work in colorectal cancer screening; Xu et al. excluded 26% for inconsistent judgments in their sample who had already completed both FIT and colonoscopy [19]. Dolan et al. and Hummel et al. also report that many participants in their samples were excluded for inconsistency with 22% and 74% excluded, respectively [18, 20]. Inconsistency can be explained in several ways. Participants may have never thought about or formulated preferences for choosing a screening strategy for CRC. An individual who is still formulating preferences may not judge comparisons in a highly consistent manner. Alternatively, the online, self-guided nature of the data collection instrument made it impossible to conduct real-time checks for understanding which may have made it more difficult for participants to consistently judge the hierarchy with multiple levels. However, our pilot test with 30 Turkers indicated that we achieved adequate understanding. This could be mitigated by using tools that streamline the process with guided sessions that walk patients through the procedure’s comparisons.

Finally, we did not include out-of-pocket cost in our AHP model. While this method is consistent with other colorectal cancer screening studies in the literature that use AHP [18–20], previous work has shown that out-of-pocket cost may be an important factor that can drive preferences for a particular strategy [36, 37]. In this study, we focused on criteria for screening strategies that will be consistent across all settings where these strategies are offered. Costs could be individually calculated and incorporated into the AHP model as its own criteria or a sub-criteria of features **of the test** if this hierarchy were implemented in a specific clinical setting,

### Implications and future directions

In this study, we found differences in the preferences for screening strategies by age. This information has policy implications and, with further evidence, could be used to inform current screening programs targeting people under age 50. Of note, participants aged 50 and under, preferred the screening strategy with the highest test effectiveness but also highest associated healthcare costs even though these individuals have the lowest age-based risk. This finding aligns with the concerns of some opponents of widening the screening age [38, 39] and warrants further attention when considering how to allocate limited resources, especially if resources must be diverted from populations with the highest absolute risks (those over 50) [40]. Future work should explore how differences in preferences for screening strategies between age groups might affect the overall health system and societal costs associated with including younger individuals in colorectal cancer screening programs.

A key component to shared decision making is that an individual’s preferences are elicited and incorporated into the decision and there is evidence to suggest that eliciting preferences increases intentions to seek screening and overall satisfaction with the colorectal cancer screening decision-making process [34, 41]. This is especially important for colorectal cancer screening because dissatisfaction with the decision making process might mean that individuals do not follow through with the recommended screening intervals that are critical to each strategy. In a healthcare setting, an AHP activity would present a high degree of burden to patients and clinicians with limited appointment time. However, an at-home, AHP activity similar to the one conducted in this study could be used to assist with the decision-making process through formulation of preferences for a particular screening strategy. Such an intervention would be strengthened by using avatars or ‘virtual humans’ to guide patients through the AHP procedure [42, 43]. Doctors or practitioners can use the information derived from such an activity to address patient understanding or target appropriate information to the things that matter most to individuals.

## Conclusions

In this study, we highlight potential differences in preferences for colorectal cancer screening strategies for individuals under age 50. To our knowledge, this is the first study designed with the intended purpose of exploring whether people under 50 think differently about preferences for CRC screening strategies than individuals over age 50. Researchers, practitioners, and policymakers should consider these results as people in younger age groups are incorporated into CRC screening programs.

## Supplementary Information

**Additional file 1.**

## Data Availability

The datasets used and/or analyzed during the current study are available from the corresponding author on reasonable request.

## Abbreviations

AHP: Analytic Hierarchy Process
CRC: Colorectal Cancer
CTC: Computed tomography colonography
FIT: Fecal immunochemical test
MCDA: Multicriteria Decision Analysis
USPSTF: United States Preventive Services Task Force

## References

[1] Wolf AMD, Fontham ETH, Church TR (2018). Colorectal cancer screening for average-risk adults: 2018 guideline update from the American Cancer Society. CA Cancer J Clin.

[2] Edwards BK, Ward E, Kohler BA (2010). Annual report to the nation on the status of cancer, 1975-2006, featuring colorectal cancer trends and impact of interventions (risk factors, screening, and treatment) to reduce future rates. Cancer..

[3] Araghi M, Soerjomataram I, Bardot A (2019). Changes in colorectal cancer incidence in seven high-income countries: a population-based study. Lancet Gastroenterol Hepatol.

[4] >Siegel RL, Miller KD, Sauer AG, et al. Colorectal cancer statistics, 2020. CA Cancer J Clin. 2020;70(3):145–64. 10.3322/caac.21601. 10.3322/caac.21601 32133645

[5] Siegel RL, Fedewa SA, Anderson WF, et al. Colorectal Cancer Incidence Patterns in the United States, 1974–2013. J Natl Cancer Inst. 2017;109(8). 10.1093/jnci/djw322. 10.1093/jnci/djw322 PMC6059239 28376186

[6] Davidson KW, Barry MJ, US Preventive Services Task Force (2021). Screening for colorectal cancer: US Preventive Services Task Force recommendation statement. JAMA.

[7] Piscitello A, Edwards DK. Estimating the Screening-Eligible Population Size, aged 45 to 74, at Average Risk to Develop Colorectal Cancer in the United States. Cancer Prev Res. 2020;13(5):443–8. 10.1158/1940-6207.CAPR-19-0527. 10.1158/1940-6207.CAPR-19-0527 32029430

[8] Lin JS, Piper MA, Perdue LA (2016). Screening for colorectal Cancer: updated evidence report and systematic review for the US Preventive Services Task Force. JAMA..

[9] Preventive Services Task Force US, Bibbins-Domingo K, Grossman DC (2016). Screening for colorectal Cancer: US Preventive Services Task Force recommendation statement. JAMA..

[10] Lee SJ, O’Leary MC, Umble KE, Wheeler SB (2018). Eliciting vulnerable patients’ preferences regarding colorectal cancer screening: a systematic review. Patient Prefer Adherence.

[11] Wortley S, Wong G, Kieu A, Howard K (2014). Assessing stated preferences for colorectal cancer screening: a critical systematic review of discrete choice experiments. Patient..

[12] Ghanouni A, Smith SG, Halligan S (2013). Public preferences for colorectal cancer screening tests: a review of conjoint analysis studies. Expert Rev Med Devices.

[13] Marshall D, McGregor SE, Currie G (2010). Measuring preferences for colorectal Cancer screening: what are the implications for moving forward?. The Patient.

[14] Harker PT, Golden BL, Wasil EA, Harker PT (1989). The art and science of decision making: the analytic hierarchy process. The analytic hierarchy process: applications and studies.

[15] Schmidt K, Aumann I, Hollander I, et al*.* Applying the Analytic Hierarchy Process in healthcare research: A systematic literature review and evaluation of reporting. BMC Med Inform Decis Mak. 2015;15:112. 10.1186/s12911-015-0234-7. 10.1186/s12911-015-0234-7 PMC4690361 26703458

[16] Liberatore MJ, Nydick RL (2008). The analytic hierarchy process in medical and health care decision making: a literature review. Eur J Oper Res.

[17] Dolan JG (1995). Are patients capable of using the analytic hierarchy process and willing to use it to help make clinical decisions?. Med Decis Mak.

[18] Dolan JG, Boohaker E, Allison J, Imperiale TF (2013). Patients’ preferences and priorities regarding colorectal cancer screening. Med Decis Mak Int J Soc Med Decis Mak..

[19] Xu Y, Levy BT, Daly JM, et al*.* Comparison of patient preferences for fecal immunochemical test or colonoscopy using the analytic hierarchy process. BMC Health Serv Res. 2015;15:175. 10.1186/s12913-015-0841-0. 10.1186/s12913-015-0841-0 PMC4411789 25902770

[20] Hummel JM, Steuten LGM, Groothuis-Oudshoorn CJM, Mulder N, Ijzerman MJ (2013). Preferences for colorectal cancer screening techniques and intention to attend: a multi-criteria decision analysis. Appl Health Econ Health Policy Auckl.

[21] Amazon. Amazon Mechanical Turk. https://www.mturk.com/. Accessed 15 Mar 2019.

[22] Casler K, Bickel L, Hackett E (2013). Separate but equal? A comparison of participants and data gathered via Amazon’s MTurk, social media, and face-to-face behavioral testing. Comput Hum Behav.

[23] Qualtrics. Provo: Qualtrics; 2005. https://www.qualtrics.com.

[24] Chew LD, Bradley KA, Boyko EJ (2004). Brief questions to identify patients with inadequate health literacy. Fam Med.

[25] Fagerlin A, Zikmund-Fisher BJ, Ubel PA, Jankovic A, Derry HA, Smith DM (2007). Measuring numeracy without a math test: development of the subjective numeracy scale. Med Decis Mak Int J Soc Med Decis Mak..

[26] Saaty TL (2003). Decision-making with the AHP: why is the principal eigenvector necessary. Eur J Oper Res.

[27] Alonso JA, Lamata MT (2006). Consistency in the analytic hierarchy process: a new approach. Int J Uncertain Fuzziness Knowl-Based Syst.

[28] IBM SPSS Statistics for Windows. Version 25.0. Armonk: IBM; 2017.

[29] Definitive Pro™. Version 3.2. Reston: Definitive Business Solutions, Inc.; 2016. https://www.definitiveinc.com/definitive-pro/.

[30] Jones RM, Devers KJ, Kuzel AJ, Woolf SH (2010). Patient-reported barriers to colorectal Cancer screening. Am J Prev Med.

[31] DeBourcy AC, Lichtenberger S, Felton S, Butterfield KT, Ahnen DJ, Denberg TD (2008). Community-based preferences for stool cards versus colonoscopy in colorectal cancer screening. J Gen Intern Med.

[32] Cho Y-H, Kim DH, Cha JM (2017). Patients’ preferences for primary colorectal cancer screening: a survey of the National Colorectal Cancer Screening Program in Korea. Gut Liver.

[33] Calderwood AH, Wasan SK, Heeren TC, Schroy PC (2011). Patient and provider preferences for colorectal Cancer screening: how does CT Colonography compare to other modalities?. Int J Cancer Prev.

[34] Schroy PC, Emmons K, Peters E (2011). The impact of a novel computer-based decision aid on shared decision-making for colorectal Cancer screening: a randomized trial (running head: SDM for CRC screening). Med Decis Mak Int J Soc Med Decis Mak.

[35] Ruffin MT, Creswell JW, Jimbo M, Fetters MD (2009). Factors influencing choices for colorectal Cancer screening among previously unscreened African and Caucasian Americans: findings from a triangulation mixed methods investigation. J Community Health.

[36] Griffith JM, Lewis CL, Brenner AR, Pignone MP (2008). The effect of offering different numbers of colorectal cancer screening test options in a decision aid: a pilot randomized trial. BMC Med Inform Decis Mak..

[37] Pignone M, Bucholtz D, Harris R (1999). Patient preferences for Colon Cancer screening. J Gen Intern Med.

[38] Bretthauer M, Kalager M, Weinberg DS (2018). From colorectal cancer screening guidelines to headlines: beware!. Ann Intern Med.

[39] Liang PS, Allison J, Ladabaum U (2018). Potential intended and unintended consequences of recommending initiation of colorectal Cancer screening at age 45 years. Gastroenterology..

[40] Ladabaum U, Mannalithara A, Meester RGS, Gupta S, Schoen RE. Cost-effectiveness and National effects of initiating colorectal cancer screening for average-risk persons at age 45 years instead of 50 years. Gastroenterology. 2019;157(1):137–48. 10.1053/j.gastro.2019.03.023 PMC7161092 30930021

[41] Christy SM, Rawl SM (2013). Shared decision-making about colorectal cancer screening: a conceptual framework to guide research. Patient Educ Couns.

[42] Ma T, Sharifi H, Chattopadhyay D (2019). Virtual Humans in Health-Related Interventions: A Meta-Analysis. Extended Abstracts of the 2019 CHI Conference on Human Factors in Computing Systems. CHI EA ‘19. Association for Computing Machinery.

[43] Apergi LA, Bjarnadottir MV, Baras JS (2021). Voice Interface technology adoption by patients with heart failure: pilot comparison study. JMIR MHealth UHealth.

