# Correction to: Evaluating preferences for colorectal cancer screening in individuals under age 50 using the Analytic Hierarchy Process

**DOI:** 10.1186/s12913-021-06924-0

**Published:** 2021-09-29

**Authors:** Travis Hyams, Bruce Golden, John Sammarco, Shahnaz Sultan, Evelyn King-Marshall, Min Qi Wang, Barbara Curbow

**Affiliations:** 1 Department of Behavioral and Community Health, School of Public Health, University of Maryland, College Park, USA; 2 Division of Cancer Control and Population Sciences, Office of the Director, National Cancer Institute, Bethesda, USA; 3 Department of Decision, Operations, and Information Technologies, Robert H. Smith School of Business, University of Maryland, College Park, USA; 4 Definitive Business Solutions, Inc., 11,921 Freedom Drive, Suite 550, Reston, VA 20190 USA; 5 Department of Medicine, Division of Gastroenterology, Hepatology, and Nutrition, University of Minnesota, Minneapolis, USA

**Correction to: BMC Health Serv Res 21, 754 (2021)**

**https://doi.org/10.1186/s12913-021-06705-9**

Following publication of the original article [1], some content was missing in Funding section. The updated Funding is given below and the missing content have been highlighted in bold typeface.

The original article [1] has been corrected.
