# Supplementary material for: Evaluating preferences for colorectal cancer screening in individuals under age 50 using the Analytic Hierarchy Process
Source: BMC Health Serv Res. 2021 Jul 29;21:754. doi: 10.1186/s12913-021-06705-9 (PMC8320058; doi:10.1186/s12913-021-06705-9)
Supplement: Supplementary file 1 — Additional file 1. [file 12913_2021_6705_MOESM1_ESM.docx]

Supplemental Materials 1:

1. **Information about colorectal cancer and screening for colorectal cancer**

“Here is some information about colorectal cancer that we would like you to read:

- Colorectal cancer occurs when cell overgrowth occurs in the parts of the large intestine known as the colon or rectum.
- Development of colorectal cancer begins as a noncancerous growth or precancerous lesion, called polyps.
- Polyps are found in up to 30 to 40% of people by age 60 but only ~10% of polyps progress to cancer.
- However, over 95% of colorectal cancers begin as polyps.
- Polyps can develop into cancer over a period of 10 to 20 years.
- This is why screening for colorectal cancer is important
- **We are now going to have you read about 3 options for colorectal cancer screening and answer questions about your preferences**”

1. **Descriptions of screening strategies**
2. **Test effectiveness:**

| **Colonoscopy…** | |
| --- | --- |
| **Effectiveness** | As a screening plan can reduce mortality by up to 85% over your lifetime |
| **Prevention** | Can detect polyps before they turn into cancer |
| **Removal** | Can remove polyps or cancer during the procedure |
| **False-positive** | Very unlikely to have an abnormal test if you do not have polyps or cancer |

| **Fecal immunochemical test (FIT)…** | |
| --- | --- |
| **Effectiveness** | As a screening plan can reduce mortality by up to 78% over your lifetime |
| **Prevention** | Can detect polyps before they turn into cancer if they are bleeding |
| **Removal** | Cannot remove polyps or cancer as part of the procedure |
| **False-positive** | Possible to have an abnormal test even if you do not have polyps or cancer |

| **Computed tomography colonography (CTC)…** | |
| --- | --- |
| **Effectiveness** | As a screening plan can reduce mortality by up to 80% over your lifetime |
| **Prevention** | Can detect polyps before they turn into cancer |
| **Removal** | Cannot remove polyps or cancer as part of the procedure |
| **False-positive** | Possible to have an abnormal test even if you do not have polyps or cancer |

*Note: Mortality reduction estimates are derived from the CISnet modeling studies (participants not provided this reference)

*Knudsen AB*, *Zauber AG*, *Rutter CM*, et al. *Estimation of benefits, burden, and harms of colorectal cancer screening strategies: modeling study for the US Preventive Services Task Force*. *JAMA.* *2016*;*315*:*2595*‐*2609*.

1. **Screening plan:**
2. *Follow up possibility:*

| **Colonoscopy…** | |
| --- | --- |
| **Follow up procedure** | No additional follow up procedures are required |
| **Polyps** | If polyps are found, they are removed and examined for cancer |
| **Cancer** | If cancer is suspected you will be referred to a specialist |

| **Fecal immunochemical test (FIT)…** | |
| --- | --- |
| **Follow up procedure** | If the test is not normal and blood is detected in your stool, you must get a follow up colonoscopy |
| **Polyps** | This test cannot confirm polyps, only a colonoscopy can confirm polyps |
| **Cancer** | This test cannot confirm cancer, only a colonoscopy can confirm cancer |

| **Computed tomography colonography (CTC)…** | |
| --- | --- |
| **Follow up procedure** | If your test is not normal, you must get a colonoscopy |
| **Polyps** | This test cannot confirm polyps, only a colonoscopy can confirm polyps |
| **Cancer** | This test cannot confirm cancer, only a colonoscopy can confirm cancer |

1. *Frequency of testing:*

| **Colonoscopy…** | |
| --- | --- |
| **Normal result** | You should be tested again in **10 years** |

| **Fecal immunochemical test (FIT)…** | |
| --- | --- |
| **Normal result** | You should be tested again in **1 year** |

| **Computed tomography colonography (CTC)…** | |
| --- | --- |
| **Normal result** | You should be tested again in **5 years** |

1. **Features of the test:**
2. *Possibility for complications:*

| **Colonoscopy…** | |
| --- | --- |
| **Minor risks** | Abdominal discomfort, cramps, bloating or gas, and minor bleeding |
| **Serious complications** | Rare and include: major bleeding (requiring hospitalization or transfusion), adverse reaction to sedation, and perforation (tearing a hole) in your colon |
| **Increased risk** | Risk goes up when more polyps are removed and as you get older |

| **Fecal immunochemical test (FIT)…** | |
| --- | --- |
| **Minor risks** | Uneasiness or anxiety from handling stool |
| **Serious complications** | Has no serious complications |
| **Increased risk** | None |

| **Computed tomography colonography (CTC)…** | |
| --- | --- |
| **Minor risks** | Cramps, bloating or gas |
| **Serious complications** | Extremely rare and include: potential development of radiation related cancer |
| **Increased risk** | Risk goes up the more times you have the test |

1. *Convenience:*

| **Colonoscopy…** | |
| --- | --- |
| **Where** | Must be done at a doctors office |
| **Driver** | You must have somebody to drive you home |
| **Work** | You must take a day off of work |

| **Fecal immunochemical test (FIT)…** | |
| --- | --- |
| **Where** | Is done at home |
| **Driver** | Does not require somebody to drive you home |
| **Work** | Does not require you to take a day off work |

| **Computed tomography colonography (CTC)…** | |
| --- | --- |
| **Where** | Must be done at a doctors office |
| **Driver** | Does not require somebody to drive you home |
| **Work** | Does not require you to take a day off work |

1. *Preparation:*

| **Colonoscopy…** | |
| --- | --- |
| **Diet** | No food or liquids starting the day before your test |
| **Laxative** | Drink a total of 64oz of laxative medication starting the day before your test to clean out your colon |
| **Bowel cleanliness** | Laxatives will make you use the restroom until your bowels are clear |

| **Fecal immunochemical test (FIT)…** | |
| --- | --- |
| **Diet** | There are no dietary restrictions |
| **Laxative** | There is no laxative medication |
| **Bowel cleanliness** | You do not need clear bowels |

| **Computed tomography colonography (CTC)…** | |
| --- | --- |
| **Diet** | No food or liquids starting the day before your test |
| **Laxative** | Drink a total of 64oz of laxative medication starting the day before your test to clean out your colon |
| **Bowel cleanliness** | Laxatives will make you use the restroom until your bowels are clear |

1. *Procedure:*

| **Colonoscopy…** |  |
| --- | --- |
| **Method** | Performed by a doctor who inserts a scope is into your anus to view the entire colon |
| **Sedation** | Sedation is given to reduce or eliminate discomfort |
| **Time for procedure** | 20-30 minutes for the procedure |
| **Recovery** | A recovery period (approximately 30-60 minutes) is necessary because of the sedation you receive |
| **Purpose** | Allows a doctor to visualize the lining of the whole colon to look for polyps or cancer |

| **Fecal immunochemical test (FIT)…** | |
| --- | --- |
| **Method** | Requires you to use a mini brush to collect stool into a tube at home that you mail in for testing |
| **Sedation** | No sedation |
| **Time for procedure** | 5 minutes |
| **Recovery** | No recovery |
| **Purpose** | Detects microscopic blood in your stool which may be from polyps or cancer |

| **Computed tomography colonography (CTC)…** | |
| --- | --- |
| **Method** | Performed by a doctor and requires that you to lay in a CT scanner after drinking a contrast liquid and having air pumped into your anus using a small tube |
| **Sedation** | No sedation |
| **Time for procedure** | 10-20 minutes |
| **Recovery** | No recovery |
| **Purpose** | Allows a doctor to use a CT scanner to make pictures of your whole colon and detect any polyps or cancer |

Supplemental Materials 2: Concordance Analysis

| N=239 | | AHP Derived Preferences | | |
| --- | --- | --- | --- | --- |
|  |  | Colonoscopy | CTC | FIT |
| Stated Pref. | Colonoscopy | 80 | 10 | 22 |
|  | CTC | 9 | 7 | 13 |
|  | FIT | 27 | 4 | 56 |
|  | I would not choose any | 5 | 0 | 6 |
